# Account of a Phrenological Visit to the Penitentiary for Young Criminals

**Published:** 1842-09

**Authors:** 


*Account of a Phrenological Visit to the Penitentiary for young Criminals at Paris, made by* M. VOISIN, *in Company with a Committee of Members of the Royal Academy of Medicine, on February* 17, 1839.—In addition to M. Voisin and the committee from the Academy of Medicine, there were present MM. Boullon and Pontignac de Villars, of whom the former was governor of the prison, the latter, secretary. Four hundred young criminals were examined, one by one, by M. Voisin; who, having looked at the form of each one's head, and examined it with his hand, directed him to go to the *right* or the *left*, according as his character or natural endowments appeared to be good or bad. These he subsequently divided into four classes, putting the worst in the first, the best in the fourth, and arranging in the two intermediate series those who formed a sort of *juste milieu* between the others.

Of the 700 boys originally examined, 254 were selected by M. Voisin as those whose good or evil qualities were most distinctly marked. The fourth, or best class, contained only 25, or one tenth of the whole; while 61 were arranged in the first or worst class. Of the remaining 168, 77 were placed in the third class, 91 in the second, the bad again preponderating.

M. Boullon, then gave his evidence as to the character of the youths thus classified by M. Voisin. He stated that M. Voisin's first class included, in a very great proportion, the bad characters in the house, or those whose intellectual faculties were most limited. The second and third divisions appeared to M. Boullon not to offer any striking differences between each other; but the fourth class comprehended almost all those children who were most docile, most intelligent, and most industrious. This class included the greater number of those who were employed as monitors in the school, or as overlookers in the workshops. The testimony of M. de Villars corresponded almost completely with that of M. Boullon.

A long discussion followed the reading of the report in the Academy. The two chief objections raised by the debaters were, that the testimony of the governor and secretary of the gaol was given after M. Voisin had pronounced on the characters of the boys, instead of before he had expressed his opinion; and secondly, that M. Voisin's classification implies that the intellectual and moral faculties are intimately connected, and become developed in the same proportion, while in reality no such absolute relation between mental and moral endowments exists.—*Bulletin de l'Académie Royal, Novembre,* 1841.

